# Pulsed electric field treatment of seeds altered the endophytic bacterial community and promotes early growth of roots in buckwheat

**DOI:** 10.1186/s12866-023-02943-5

**Published:** 2023-10-13

**Authors:** Hao Qu, Yi Wang, Baijuan Wang, Chengyun Li

**Affiliations:** 1 State Key Laboratory for Conservation and Utilization of Bio-Resources in Yunnan, Yunnan Agricultural University, Kunming, China; 2 College of Tea Science, Yunnan Agricultural University, Kunming, China; 3 Tea Research Institute, Yunnan Academy of Agricultural Sciences, Menghai, China; 4 Yunnan-CABI Joint Laboratory for Integrated Prevention and Control of Trans-boundary Pests, Yunnan Agricultural University, Kunming, China

**Keywords:** Buckwheat, Pulsed electric field, Endophytic, Bacteria, Root

## Abstract

**Background:**

Endophytic bacteria provide nutrients and stimulate systemic resistance during seed germination and plant growth and development, and their functional properties in combating various stresses make them a powerful tool in green agricultural production. In this paper we explored the function of the endophyte community in buckwheat seeds in order to provide a theoretical basis for the application and scientific research of endophytes in buckwheat cultivation. We used pulsed electric field (PEF) technology to treat buckwheat seeds, monitored the effect of high-voltage pulse treatment on buckwheat seed germination, and analyzed the diversity of endophytic bacteria in buckwheat seeds using the amplicon sequencing method.

**Results:**

PEF treatment promoted root development during buckwheat seed germination. A total of 350 Operational taxonomic units (OTUs) that were assigned into 103 genera were obtained from control and treatment groups using 16SrRNA amplicon sequencing technology. Additionally, PEF treatment also caused a significant decrease in the abundance of Actinobacteria, Proteobacteria, and Bacteroidetes. The abundance of 28 genera changed significantly as well: 11 genera were more abundant, and 17 were less abundant. The number of associated network edges was reduced from 980 to 117, the number of positive correlations decreased by 89.1%, and the number of negative correlations decreased by 86.6%.

**Conclusion:**

PEF treatment promoted early root development in buckwheat and was able to alter the seed endophytic bacterial community. This study thus makes a significant contribution to the field of endophyte research and to the application of PEF technology in plant cultivation.

## Introduction

Plant endophytic bacteria communities are rich in diversity and provide a treasure trove of microbial resources to the plants themselves. Endophytic bacteria promote plant health when plants are stressed abiotically or biotically and thus help plants cope better with environmental stress [1]. Endophytic bacteria also play an important role in seed germination and plant growth and development, and their functional properties in combating multiple stresses by providing nutrients or stimulating systemic disease resistance make them a powerful tool in green agricultural practices [2]. The main types of plant endophytes were Firmicutes, Bacteroidetes, Proteobacteria, and Actinobacteria, but the core community structure of and roles they play differ between plants [3–5].

In recent years emerging technologies such as cold plasma, pulsed electric fields (PEF), ultrasound, and chemical methods have been applied to the endophyte research [6–8]. Research on the electro-biological effects generated by PEF actually began in the 18th century. The first researchers to do this treated more than 20 varieties of seeds such as barley, corn, rice, cotton, and rapeseed with PEF, and the effects of PEF on seed viability, seed germination, seedling growth, and plant development were analyzed in turn [9]. They found that PEF treatment had significant effects in improving seed germination rate, inhibiting seed degradation, shortening plant growth cycle, increasing yield, and enhancing plant stress resistance.

The inhibitory and stimulating effects of electric fields on seed growth are related to the physiological state, electric field intensity, and intensity of the seeds, resulting in significant biochemical and physiological differences involved in the various methods of seed germination and swelling. However, PEF technology can be used to manipulate all of these processes [10]. PEF treatment can improve water absorption rate, germination rate, and seedling growth of wheat seeds, and the total phenols, chlorophyll, carotenoids, soluble proteins, minerals, and amino acids in wheat seeds treated with PEF have been shown to be significantly elevated as well [11]. As of this writing PEF has not been used in the study of plant endophytes. With this in mind we set out to apply PEF methods to buckwheat seeds and discovered a phenomenon whereby PEF can effectively remove endophytic bacteria from the seeds. In addition, expedited root growth was promoted when the PEF intensity was 1kv/cm for 1 h. We also used 16 S rRNA sequencing to analyze the response of the endophytic bacteria in the buckwheat seeds to pulsed electrical fields and found that these pulses altered the structure of the endophytic bacterial community of the seeds. The bacterial taxa became differentiated in response to PEF. This study thus provides a new approach to the application of PEF technology in the field of endophyte function research.

## Materials and methods

### Samples collection

This study was carried out in accordance with the relevant national/institutional guidelines. The plant material was purchased from the “Hongqiaodi” seed company from Yunnan, China. Common buckwheat (*Fagopyrum tataricum*) seeds were harvested in 2020 for experiments conducted between 2020 and 2021, and the experiments were divided into control (CK) and pulsed electric field (PEF) treatments.

### Seed treatment with PEF

**Fig. 1:**
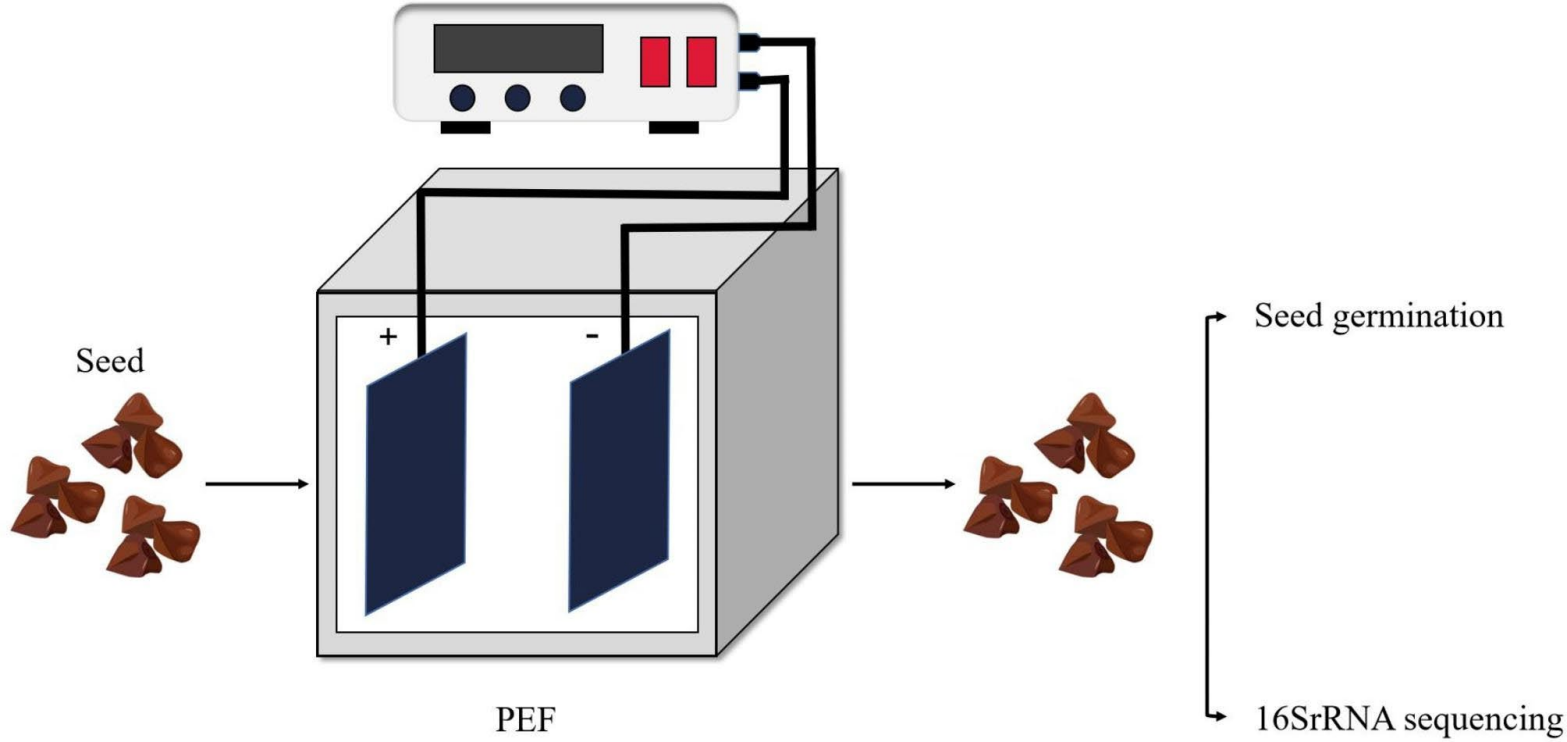
Schematic view of seed treatment with PEF. Buckwheat seeds were treated in containers with high-voltage pulsed electrodes, and then seed germination experiments and 16SrRNA sequencing were performed

A PEF power supply was used to connect positive and negative electrodes in a glass box of 40 cm × 60 cm to form an adjustable electric field device capable of generated uniformly distributed electric discharges (Fig. 1). The seeds were placed in a PEF at room temperature, with electric field intensity, frequency, and duty ratio of 1 kV/cm, 120 Hz, and 70%, respectively, for 1 h. The treated buckwheat seeds were then used for 16 S rRNA sequencing and seed germination experiments. The seed germination experiments were carried out in filter paper petri dishes, with 3 ml of added deionized water, and germination indicators were collected on the third day. All experiments were repeated 3 times. Eighty seeds for each of 3 independent experiments were used for each experimental group.

Research has shown that excessive electric field intensity can kill microorganisms, so experiments on the promotion of seed activity using PEF use only low intensity, high-voltage pulses. Therefore, we selected an electric field intensity of 1 kV/cm for our experiment. To establish an optimum PEF treatment duration, buckwheat seeds were treated for 1, 2, or 4 h of cumulative treatment duration, though our results indicated that treatment timeout could not promote seedling root growth.

### 16SrRNA sequencing and analysis

Using primers 341 F and 806R, amplification of the bacterial 16 S rRNA gene v3-v4 region was performed. PCR testing was then conducted using specific primers with Barcode, Phusion®, and High-Fidelity PCR Master Mix with GC Buffer from New England Biolabs, and high-efficiency high-fidelity enzymes according to the selection of the sequencing region. The amplification procedure was as follows: pre-denaturation at 95℃for 5 min and 35 cycles (denaturation at 95℃for 45 s, retreatment at 55℃ for 45 s, extension at 72℃ for 90 s), followed by stable extension at 72 °C for 7 min, and all PCR products were detected by electrophoresis on a 2% agarose gel. Qualified PCR products were purified by magnetic beads, quantified by enzyme labeling, and samples were mixed in equal amounts according to the concentration of PCR products. After thorough mixing, we used 2% agarose gel electrophoresis to detect the PCR products and used the gel recovery kit provided by Qiagen to recover the products of the target bands. According to the characteristics of the amplified 16 S region, a small fragment library was then constructed, and paired-end sequencing was performed on the library based on the Illumina NovaSeq sequencing platform. Finally, splicing and filtering, OTUs clustering, species annotation, and abundance analysis were all carried out.

### Analysis of alpha and beta diversity index

The data of each sample was homogenized using the sample with the least amount of data as the standard for homogenization. Alpha diversity analysis and beta diversity analysis were then conducted based on the homogenized data. The significance of differences between samples from different compartments was tested using the Kruskal-Wallis test (*p*-value < 0.05), and the cumulative sum scaling (CSS) method was used to normalize the OTUs to estimate beta diversity. Bray-Curtis distances between samples were used for principal coordinate analysis (PCoA), with Qiime software (Version 1.9.1) used to draw PCoA diagrams. To test the impact of location and compartment on the estimated explained variance, PERMANOVA analysis was also performed.

### Bacterial correlation networks

SparCC co-occurrence analysis was performed using the R package “SpiecEasi” to calculate the correlation coefficient values between the communities. The true score was evaluated based on the Dirichlet distribution of the observed values, and then the observed score was obtained by averaging 5 estimates. Random substitution based on resampling was used to evaluate statistical significance. The SparCC algorithm begins by resampling the original dataset using the bootstrap method to obtain a random dataset. *P*-values are calculated from these random data to evaluate the significance of the initial observation scores. The correlations of the OTUs are calculated in the random dataset to obtain the correlation matrix of these random values. Then, p-values are generated again by comparing the distribution of values in the correlation matrix of observed values to the correlation matrix of random values. We used R to build a network from the adjacency matrix and convert the network format. Correlations with a magnitude > 0.5 and statistical significance (*p*-value < 0.05) were included into subsequent network analysis, where the networks were visualized in Gephi.

### Statistical analysis

The Uparse algorithm was used to cluster the effective tags of all samples, and the sequences were clustered into OTUs with 97% consistency. The Mothur method and the SSUrRNA SILVA138 database SILVA138 were used for species annotation analysis to obtain taxonomic information and classify the organisms at each taxonomic level. The differences in microbial composition between the control and treatment groups were calculated using ANOVA (*p*-value < 0.05).

## Results

### Effect of PEF treatment on seed germination and seeding root length

**Fig. 2:**
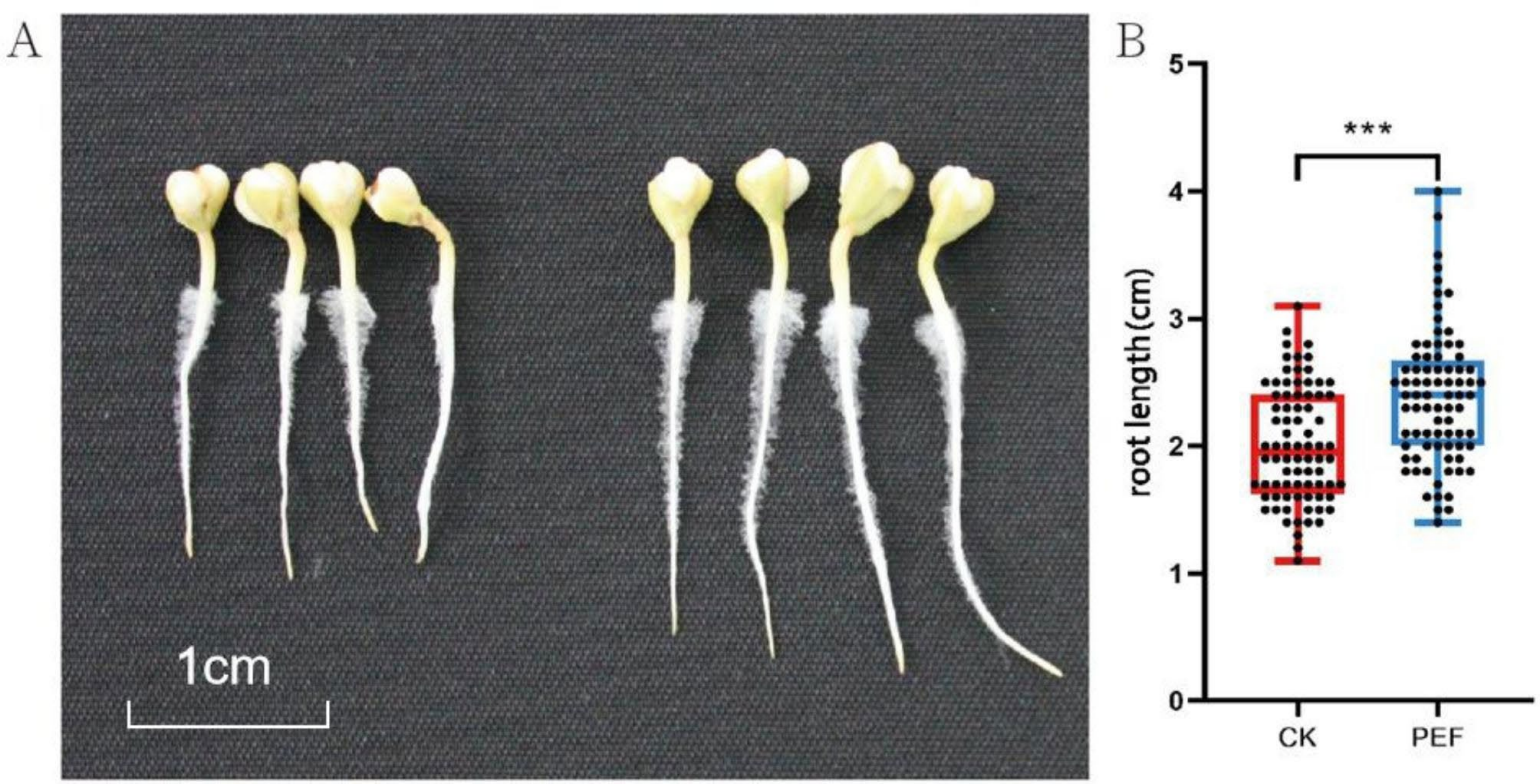
Image of buckwheat seeding cultured for three days after PEF treatment. Representative samples (**A**) and root length (**B**) of buckwheat seedlings before (CK) and after (PEF) treatment (***p<0.001)

To investigate the role of endophytic bacteria in buckwheat seeds and to explore the application of PEF technology in plant cultivation, we used PEF to treat buckwheat seeds and conducted seed germination experiments. The results indicate that the germination rate of the untreated seeds was 92.3%±2.5%, and that of the PEF-treated seeds was 91%±4%. The hundred seeding weight was 6.32 g ± 0.06 and 6.67 g ± 0.25 g, and the germination index was 47.46 ± 2.42 and 48.19 ± 1.73 for the untreated and treated groups, respectively (Table 1). The PEF did not cause statistically significant changes in germination percentage, hundred seeding weight, or germination index. However, the root length of PEF-treated germination (2.38 ± 0.14 cm) was significantly different from that of CK (2.01 ± 0.07 cm)(Fig. 2).

**Table 1:** Seed germination data

	Dry matter weight(g)	sprouting rates(%)	weight of 100 buds(g)	germination index
CK	2.206 ± 0.092	92.3 ± 2.5	6.32 ± 0.06	47.46 ± 2.42
PEF	2.216 ± 0.045	91 ± 4	6.67 ± 0.25	48.19 ± 1.73

### Effects of PEF on the diversity of endophytic bacteria

**Fig. 3:**
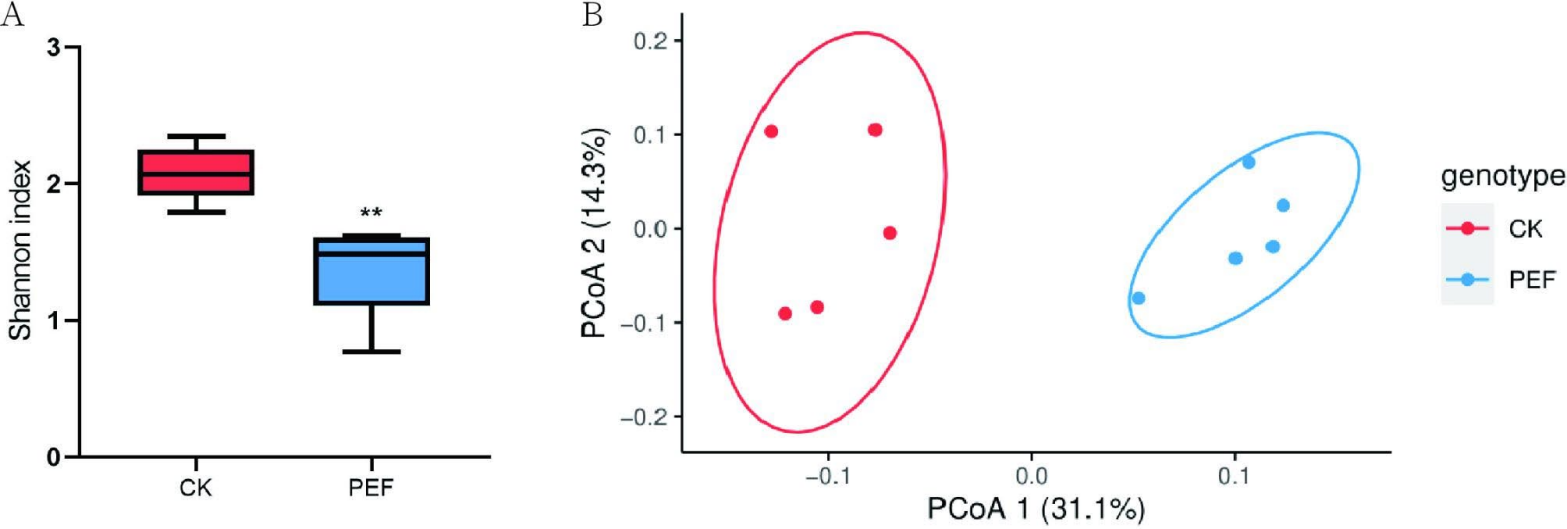
Alpha and beta diversity of bacterial community in buckwheat seeds before and after PEF treatment. (**A**) Alpha diversity measurements for microbial communities from the Buckwheat seeds (**p<0.01). (**B**) PCoA of beta diversity indicated that PEF treatment affected the bacterial community

To explore the effects of PEF on the endophytic bacterial community under the condition of promoting seeding root development, 16 S rRNA sequencing was performed using the NovaSeq 6000 platform. In total, 631,851 raw reads were generated. 619,646 clean tags were obtained, and the effective tag totals were 198,570 and 271,835 for the CK and PEF treated, respectively. The quality score (Q30) percentage was above 93%. OTUs clustering was performed on the effective tags of all samples, with 97% identification. A total of 350 bacterial OTUs were identified and assigned into 10 phyla and 103 genera. Alpha diversity analysis revealed that the endophytic bacterial Shannon index was significantly lower in the PEF treated seeds. Moreover, the PCoA of beta diversity indicated that the microflora of the CK and PEF treated seeds exhibited a clear separation (Fig. 3). Collectively, PEF exerted significant effects on the seed bacterial communities.

### Dynamics of the endophytic bacteria co-occurrence network

**Fig. 4:**
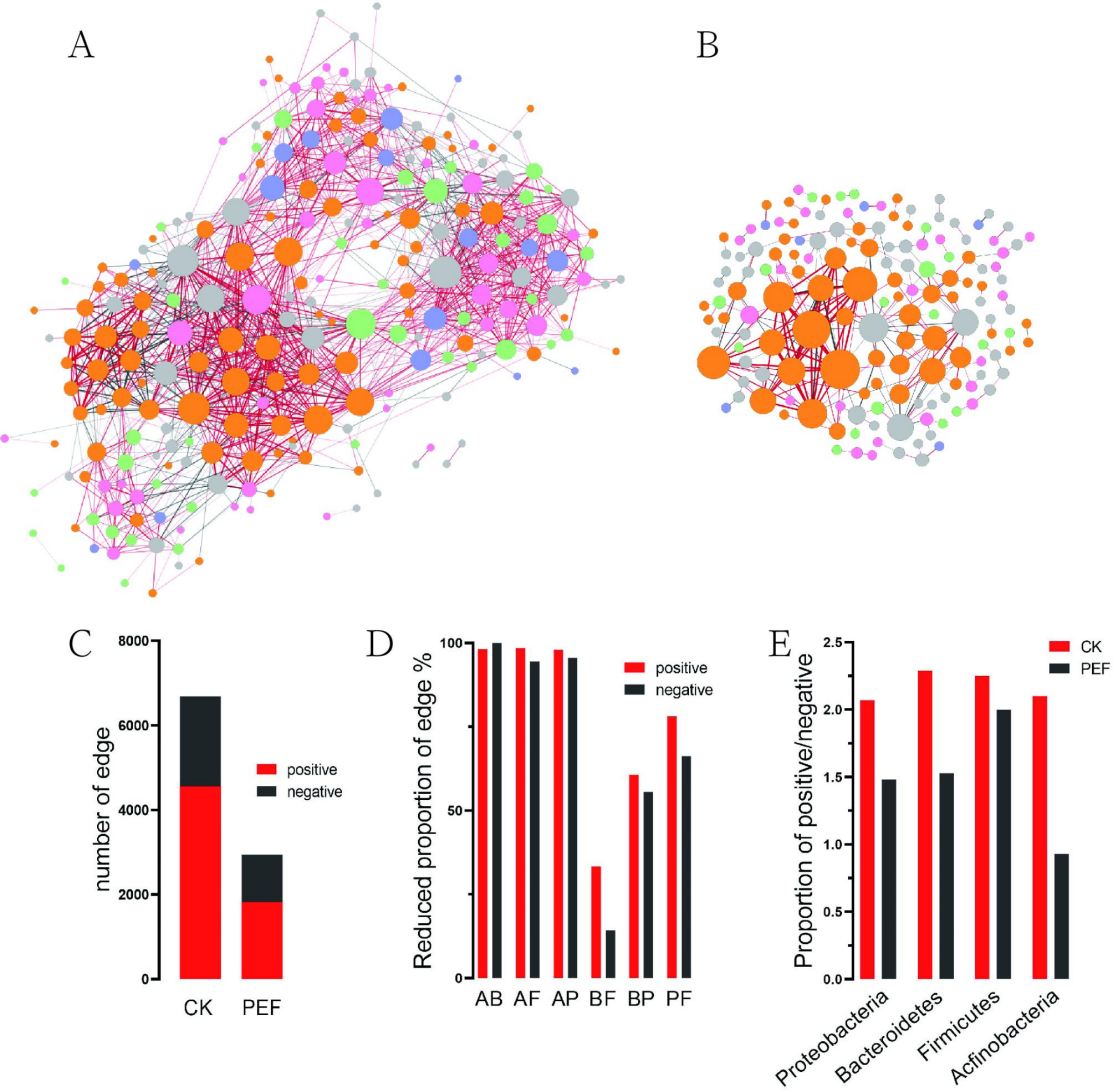
Analysis of bacterial interaction network in buckwheat seeds control (**A**) and PEF treated (**B**). The edge color represents positive (red) and negative (black) correlations. The point color represents Bacteroidetes (Blue), Actinobacteria (pink), Proteobacteria (yellow) and Firmicutes (green) correlations. The number of edges (**C**). Statistics of the number of edges of the associated network (**D**), and the X-axis represents correlation between Actinobacteria and Bacteroidetes (AB), Actinobacteria and Firmicutes (AF), Actinobacteria and Proteobacteria (AP), Bacteroidetes and Firmicutes (BF), Bacteroidetes and Proteobacteria (BP) and Proteobacteria and Firmicutes(PF). The number of control and PEF treatment edges is at the Phylum level (**E**)

Co-occurrence network analysis to evaluate the remodeling effects of PEF treated on inter-bacteria interactions. The correlation values between the OTUs using the SpiecEasi R package and constructed the co-occurrence network used Gephi. The results indicate that the untreated network had a higher level of edge connectivity compared to the PEF treated seeds. The number of network nodes was 180 for the CK, compared to 42 for the PEF treated (Fig. 4A, B). In addition, the number of edges was 980 for the CK and 117 for the PEF treated. Similarly, the number of positive correlations was 545 for the CK compared to 59 for the PEF treated, and negative correlation was 435 compared to 58 for the CK and PEF treated, respectively. Correlation results show that the proportion of positive correlations was 55.6% compared to 50.4% and that negative correlation was 44.4% compared to 49.6% for the CK and PEF treated (Fig. 4C).

To reveal the effect of PEF treatment on the main bacteria present in the community, we further analyzed them at the phylum level. The statistics for the nodes at this level showed that the number of Proteobacteria, Bacteroidetes, Actinobacteria, and Firmicutes were lower by 40, 15, 28, and 30, respectively in the CK. Specifically, positive correlations with Proteobacteria were 82.31% lower, Bacteroidetes were 100% lower, Actinobacteria were 98.31% lower, and Firmicutes were 98.7% lower in the PEF treated. The same statistics for negative correlations were 81.33%, 100%, 95.07%, 95.96%, respectively (Fig. 4D).

Furthermore, the network connectivity of the Bacteroidetes was 0 in the treatment group, though the proportion of Proteobacteria in the network was 64% compared to 97% for the untreated and treated seeds, respectively. Collectively, Actinobacteria, Bacteroidetes, and Firmicutes had the largest difference in the number of associated network edges, and Bacteroidetes had the smallest. Finally the differences in positive correlation were greater than those in negative correlation (Fig. 4E).

### Effects of PEF on endophytic bacteria composition

**Fig. 5:**
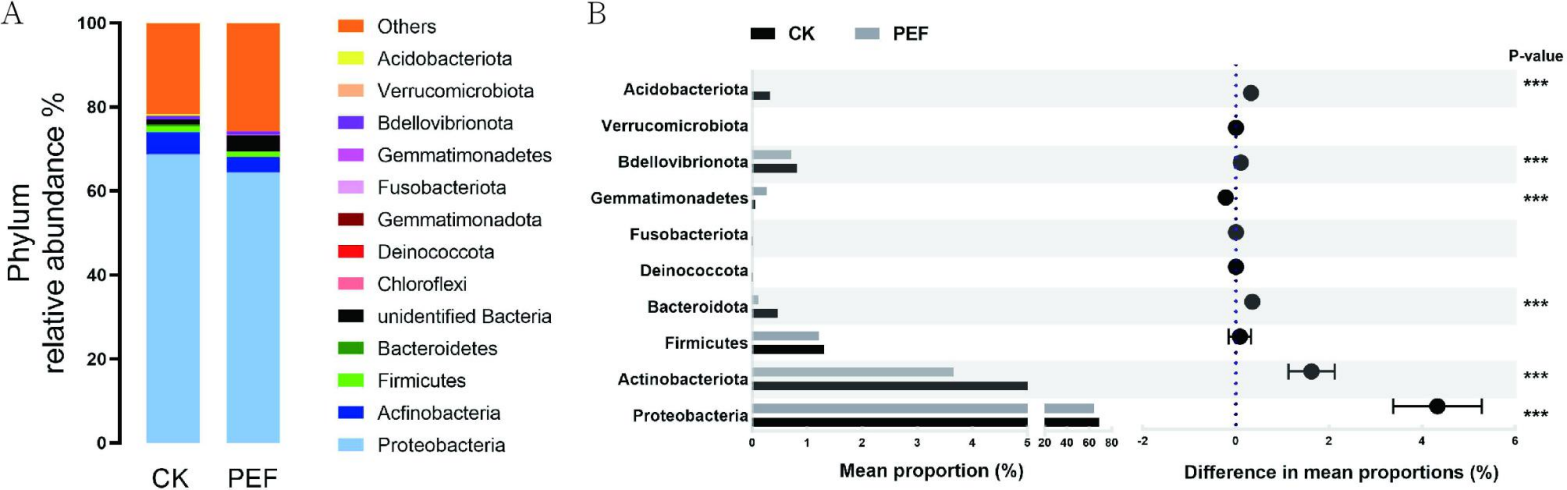
The phylum level of bacterial community in buckwheat seeds. (**A**) Relative abundances of bacterial phyla. (**B**) Significant differences in the abundances of phylum. *P* values were calculated using Student’ s t test (***p < 0.001)

Classification analysis was performed to analyze the effects of PEF treatment on the bacterial community. A total of 10 bacterial phyla were annotated across all samples (Fig. 5A). A decrease in the abundance of Actinobacteria, Proteobacteria, and Bacteroidetes was observed after PEF treatment (Fig. 5B). However, the abundance of Firmicutes, which has the thickest cell wall of these groups of bacteria, was not statistically different between groups, which may indicate that the bacterial cell wall played a role in the response to PEF.

**Fig. 6:**
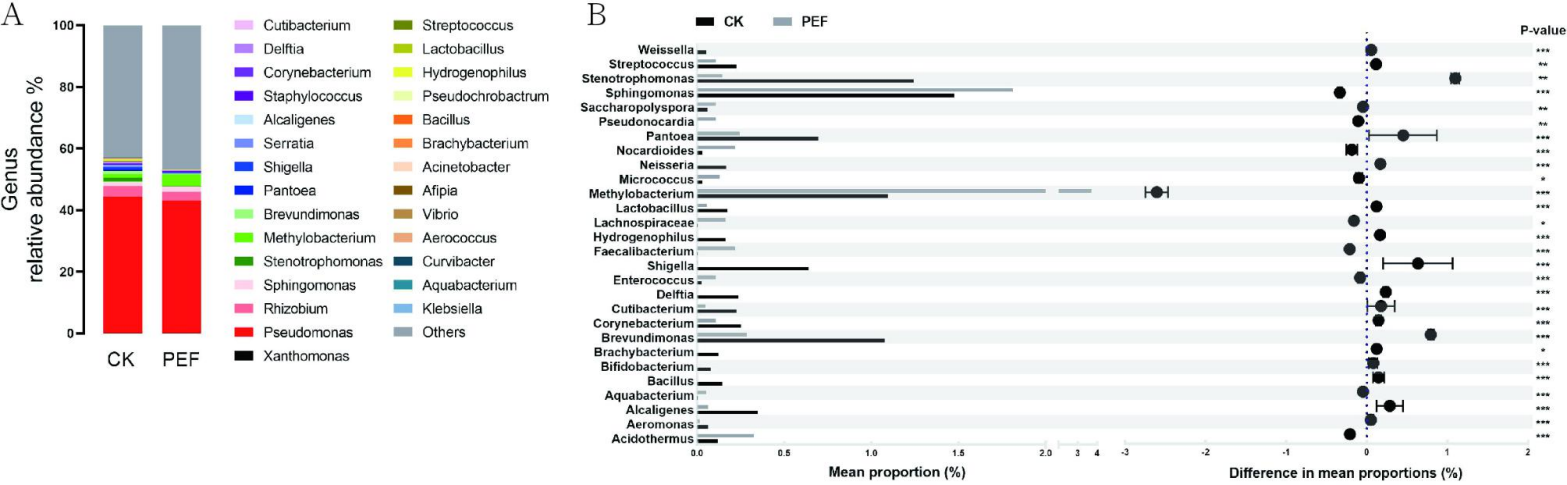
The genus level of bacterial community in buckwheat seeds. (**A**) Relative abundances of bacterial genus. (**B**) Significant differences in the abundances of genus. *P* values were calculated using Student’s t test (***p < 0.001)

At the genus level, *Pseudomonas* was the predominant genus (44.4% of total sequences) in the CK. Other OTUs belonged to the bacterial classes *Rhizobium* (3.3%), *Sphingomonas* (1.4%), *Stenotrophomonas* (1.2%), *Methylobacterium* (1%), and *Pantoea* (0.7%) (Fig. 6A). Among them, the abundance of 28 genera changed significantly after PEF treatment. Notable differences observed after PEF treatment included a large relative decrease in the abundance of *Streptococcus*, *Neisseria*, *Bifidobacterium*, *Brevibacterium*, *Delft*, *Bacillus*, and *Hydrogenobacteria* (Fig. 6B). All genera except *Bacillus* are non-sporogenic, which may provide clues to their bacterial response to PEF.

**Fig. 7:**
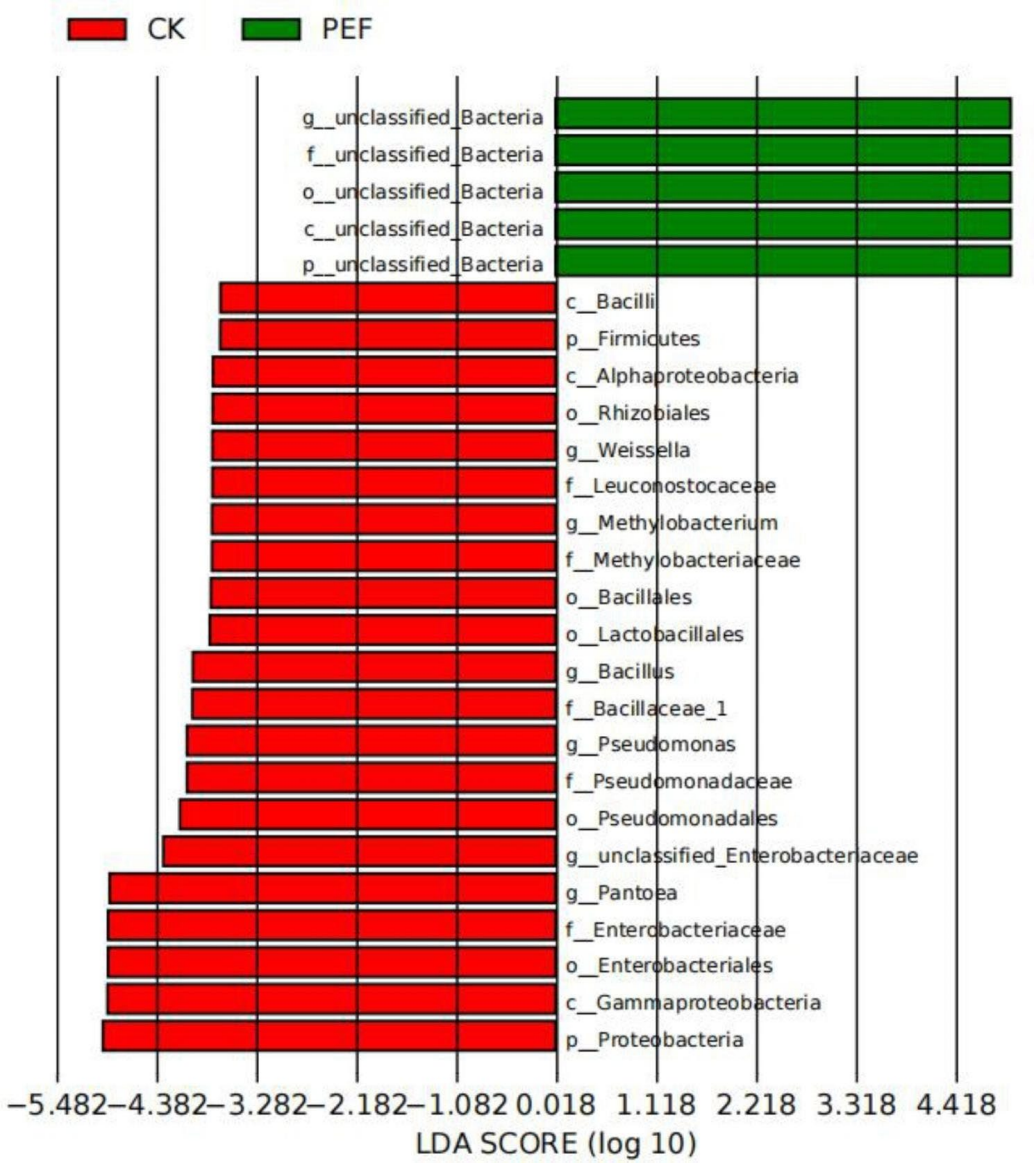
Differential abundance between CK and PEF by linear discriminant analysis (LDA) > 4. Negative LDA score represents enrichment in CK (red) and positive LDA score represents the opposite (green)

Next, we utilized the linear discriminant analysis effect size (LEfSe) algorithm, and these results revealed that *Bacillus*, *Weissella*, *Methylobacterium*, *Pantoea*, and *Pseudomonas* were consistently depleted after PEF treatment (Fig. 7). In addition, whether in the environment or in plants, the number of bacilli was greater than the number of cocci [12]. Similarly, the number of changes in the abundance of the bacilli from our treatment was much greater than that of the cocci (higher abundance: 9 bacilli, 2 cocci; lower abundance: 15 bacilli, 2 cocci) as well. In total there were 14 Gram-positive and 14 Gram-negative bacteria from 28 taxa with significant abundance changes.

## Discussion

### PEF affected the endophytic bacterial community in buckwheat seeds

Endophytes exist in various parts of plants and play an important role in plant growth and development [13]. However, existing functional studies of endophytes are very limited, because many endophytes are difficult to isolate and culture and therefore cannot be studied by directional removal of a single endophyte group [6, 14]. Recently, however, physical methods such as cold plasma, ultrasound, and PEF have been gradually applied to the study of microbial community structure and function, although little is still known about the effects of endophytes compared to the effects of physical methods such as PEF on plant growth and development.

Currently, PEF is mostly used in sterilization procedures in food processing since it can kill Escherichia coli in cider (electric field strength 80 kV/cm), Lactobacillus in beer (electric field strength 13 kV/cm), Saccharomyces cerevisiae in orange juice (35 kV/cm), and *Alternaria* and *Xanthomonas* on seed surfaces (electric field strength 12 kV/cm) [15–18]. However, these studies use high (greater than 10 kV/cm) to achieve sterilization. Here, our study did kill some buckwheat seed endophytes using a lower PEF strength (1 kV/cm), but this electric field strength has only been suggested in previous studies to promote the growth and development of plants such as wheat, sorghum, and eggplant by treating their seeds [10, 19].

Previous studies have demonstrated that PEF treatment and the absence of bacterial blooms during such treatment times can cause abundance changes that may only result in fewer distinct taxa [20]. Such studies support our claim that PEF treatment killed some bacteria, resulting in changes in bacterial community abundance. In addition, plant cells differ from bacterial cells, and such treatment conditions may not be sufficient to affect seed survival [21, 22]. Our research thus provides a basis for the killing of plant endophytes by PEF as well as a novel first step for research methods into plant endophytes.

### Different bacterial taxa respond differently to PEF

PEF treatment produces reversible and irreversible electroporation due to differences in cell membrane repair capacity [23], so differences in bacterial cell membranes are an important factor in their response to PEF. In addition, although PEF has a strong penetrative ability, we speculate that the distribution of bacteria in seeds and the difference in water content inside the seeds may affect the penetration effect of PEF on bacteria as well. Moreover, most existing studies are in liquid or food, and the bacteria are relatively active in these environments [20]. With the low water content in seeds, the state of their bacteria is still unknown, and it remains to be explored whether the differences in PEF responses are due to the state of the bacteria.

Existing studies have shown that the differences in bacterial protein abundance are consistent between certain low-intensity stress stimuli, such as cold plasma treatment, low-intensity UV-B treatment, and electromagnetic field treatment [24, 25]. However, we cannot determine the extent of the effect of PEF on various proteins in the cell membrane, so whether our observed effects of PEF treatment on bacterial taxa stem from a biological relationship or were just noise still needs further research.

This study has attempted to lay the foundation for the application of PEF technology to the detection of bacterial status and stress resistance. The PEF method can remove some endophytic bacteria while protecting the normal germination of seeds, which may spark new ideas for the study of the association between seeds and endophytes.

### Positive effects of PEF treatment on seeds

Endophytic bacteria play an important role in seed germination and plant growth and development by providing nutrients and stimulating systemic disease resistance [26, 27]. PEF treatment resulted in less microbial diversity in buckwheat seeds, primarily in Proteobacteria, Actinobacteria and Bacteroidetes. This lower abundance led to a higher abundance of some other bacteria, and the overall endophytic bacterial community in the buckwheat seeds changed. This phenomenon had no significant adverse effect on the health of the seed germination, but instead promoted root development during germination. However, PEF treatment may still affect seeds in other aspects, such as cellular metabolism [11].

The dominant phyla in buckwheat seeds are Proteobacteria, Actinobacteria, and Firmicutes, which are similar to those found in in rice, cereals, and *Arabidopsis* [], and they may play a similar functions in each of these plants. It was found that *Methylobacter* exhibited growth-promoting effects in a variety of plants [29], and other studies have shown that *Sphingomonas* have growth-promoting functions in plants as well [30,31]. Here, PEF treatment resulted in a greater abundance of *Methylobacter* and *Sphingomonas* in buckwheat seeds, and this higher abundance may be one of the reasons that the PEF promoted seed root development. In addition, among the 28 genera with significant changes in abundance in this study, *Pantoea* is the only phytopathogen-related one to be identified as such in multiple papers [32]. *Pantoea* is common in endophytes, and the treatments in this study resulted in a significantly lower abundance of it, which may have had a positive effect on seed germination. Similarly, *Pseudomonas* is quite common in the rhizosphere and plant tissues and is considered to have positive significance for plant growth and development as well [33]. Moreover, it has a dominant position in buckwheat seeds. Its core position was not changed by PEF treatment, which may also have had positive implications for seed survival.

Crucially, PEF can perforate the cell membrane and cause the migration of metal ions, which can affect plant growth and development as well as that of bacteria [19, 34]. Iron in particular is a key nutrient for the survival of most bacteria and also affects the growth and development of plants [35]. Bacteria take up iron through siderophores, and different taxa exert positive and negative effects on plants during this process [36, 37]. Under the action of PEF, the connection among iron elements, bacteria, and seeds may therefore also produce complex changes. Studies have shown that *Methylobacter* and *Sphingomonas* all have the ability to produce siderophores, and these taxa have also been shown to help host plants acquire iron [38–41]. Changes in the abundance of these taxa may thusly affect the growth and development of plants. In addition, the reduction of bacterial community diversity also reduces reduce competition for nutrients and promotes the utilization of nutrients by plants rather than bacterial communities.

One limitation to our study is that we only detected the endophytic bacterial groups in seeds after PEF treatment, and did not detect the endophytic bacterial group in the plumule. However, the study of the structure and function of the initial endophytic bacterial community in seeds also holds research significance in the field of plant growth and development.

## Conclusion

Collectively, our results show that PEF treatment promoted early root development in buckwheat and was also able to alter the seed endophytic bacterial community. This study thus makes a significant contribution to the field of endophyte research and the application of PEF technology in plant cultivation.

## Data Availability

The sequencing dataset analyzed during the current study is available in the NCBI Sequence Read Archive (PRJNA950237).

## Abbreviations

PEF: Pulsed electric field
OTU: Operational taxonomic unit
CK: Control
CSS: Cumulative sum scaling
PCoA: Principal coordinate analysis
LEfSe: Linear discriminant analysis effect size
UV-B: Ultraviolet-b radiation

## References

[1] Compant S, Clément C, Sessitsch A (2010). Plant growth-promoting bacteria in the rhizo- and endosphere of plants: their role, colonization, mechanisms involved and prospects for utilization. Soil Biol Biochem.

[2] White JF, Kingsley KL, Zhang Q, Verma R, Obi N, Dvinskikh S, Elmore MT, Verma SK, Gond SK, Kowalski KP. Review: endophytic microbes and their potential applications in crop management. Pest Manag Sci. 2019 Oct;75(10):2558–65. 10.1002/ps.5527 PMC6771842 31228333

[3] Raj G, Shadab M, Deka S, Das M, Baruah J, Bharali R, Talukdar NC. Seed interior microbiome of rice genotypes indigenous to three agroecosystems of Indo-Burma biodiversity hotspot. BMC Genomics. 2019 Dec;3(1):924. 10.1186/s12864-019-6334-5 PMC6892021 31795935

[4] Abdullaeva Y, Ambika Manirajan B, Honermeier B, Schnell S, Cardinale M. Domestication affects the composition, diversity, and co-occurrence of the cereal seed microbiota. J Adv Res 2020 Dec 17;31:75–86. 10.1016/j.jare.2020.12.008 PMC8240117 34194833

[5] Chen T, Nomura K, Wang X, Sohrabi R, Xu J, Yao L, Paasch BC, Ma L, Kremer J, Cheng Y, Zhang L, Wang N, Wang E, Xin XF, He SY. A plant genetic network for preventing dysbiosis in the phyllosphere. Nature. 2020 Apr;580(7805):653–7. 10.1038/s41586-020-2185-0 PMC7197412 32350464

[6] Tamošiūnė I, Gelvonauskienė D, Haimi P, Mildažienė V, Koga K, Shiratani M, Baniulis D. Cold plasma treatment of sunflower seeds modulates Plant-Associated Microbiome and stimulates Root and lateral organ growth. Front Plant Sci. 2020 Aug;28:11:568924. 10.3389/fpls.2020.568924 PMC7485318 32983218

[7] Wang LH, Wang MS, Zeng XA, Zhang ZH, Gong DM, Huang YB. Membrane Destruction and DNA binding of Staphylococcus aureus cells Induced by Carvacrol and its combined effect with a Pulsed Electric Field. J Agric Food Chem 2016 Aug 17;64(32):6355–63. 10.1021/acs.jafc.6b02507 27420472

[8] Muñoz R, Viveros N, Bevilacqua A, Pérez MS, Arévalo-Villena M. Effects of ultrasound treatments on wine microorganisms. Ultrason Sonochem. 2021 Nov;79:105775. 10.1016/j.ultsonch.2021.105775 PMC8517920 34649166

[9] Gonzalez ME, Barrett DM. Thermal, high pressure, and electric field processing effects on plant cell membrane integrity and relevance to fruit and vegetable quality. J Food Sci. 2010 Sep;75(7):R121–30.10.1111/j.1750-3841.2010.01763.x21535564

[10] Leong SY, Burritt DJ, Oey I. Electropriming of wheatgrass seeds using pulsed electric fields enhances antioxidant metabolism and the bioprotective capacity of wheatgrass shoots. Sci Rep. 2016 May;5:6:25306. 10.1038/srep25306 PMC4857078 27147445

[11] Ahmed Z, Manzoor MF, Ahmad N, Zeng XA, Din ZU, Roobab U, Qayum A, Siddique R, Siddeeg A, Rahaman A. Impact of pulsed electric field treatments on the growth parameters of wheat seeds and nutritional properties of their wheat plantlets juice. Food Sci Nutr. 2020 Apr;5(5):2490–500. 10.1002/fsn3.1540 PMC7215213 32405405

[12] Baquero F, Coque TM, Galán JC, Martinez JL. The origin of niches and species in the Bacterial World. Front Microbiol. 2021 Mar;17:12:657986. 10.3389/fmicb.2021.657986 PMC8010147 33815348

[13] Trivedi P, Leach JE, Tringe SG, Sa T, Singh BK. Plant-microbiome interactions: from community assembly to plant health. Nat Rev Microbiol. 2020 Nov;18(11):607–21. 10.1038/s41579-020-0412-1 32788714

[14] Song JS, Kim SB, Ryu S, Oh J, Kim DS. Emerging plasma technology that alleviates crop stress during the early growth stages of plants: a review. Front Plant Sci. 2020 Jul;15:11:988. 10.3389/fpls.2020.00988 PMC7373780 32760412

[15] Iu J, Mittal GS, Griffiths MW. Reduction in levels of Escherichia coli O157:H7 in apple cider by pulsed electric fields. J Food Prot. 2001 Jul;64(7):964–9. 10.4315/0362-028x-64.7.964 11456204

[16] Ulmer HM, Heinz V, Gänzle MG, Knorr D, Vogel RF (2002). Effects of pulsed electric fields on inactivation and metabolic activity of Lactobacillus plantarum in model beer. J Appl Microbiol.

[17] Elez-Martínez P, Escolà-Hernández J, Soliva-Fortuny RC, Martín-Belloso O. Inactivation of Saccharomyces cerevisiae suspended in orange juice using high-intensity pulsed electric fields. J Food Prot. 2004 Nov;67(11):2596–602. 10.4315/0362-028x-67.11.2596 15553647

[18] Evrendilek GA, Karatas B, Uzuner S, Tanasov I. Design and effectiveness of pulsed electric fields towards seed disinfection. J Sci Food Agric. 2019 May;99(7):3475–80. 10.1002/jsfa.9566 30623440

[19] Kral N, Hanna Ougolnikova A, Sena G. Externally imposed electric field enhances plant root tip regeneration. Regeneration (oxf). 2016 Aug 20;3(3):156–67. 10.1002/reg2.59 PMC5011479 27606066

[20] Garner AL. Pulsed electric field inactivation of microorganisms: from fundamental biophysics to synergistic treatments. Appl Microbiol Biotechnol. 2019 Oct;103(19):7917–29. 10.1007/s00253-019-10067-y 31392376

[21] Ben Ammar J, Lanoisellé JL, Lebovka NI, Van Hecke E, Vorobiev E. Impact of a pulsed electric field on damage of plant tissues: effects of cell size and tissue electrical conductivity. J Food Sci. 2011 Jan-Feb;76(1):E90–7. 10.1111/j.1750-3841.2010.01893.x 21535680

[22] Asavasanti S, Ersus S, Ristenpart W, Stroeve P, Barrett DM. Critical electric field strengths of onion tissues treated by pulsed electric fields. J Food Sci. 2010 Sep;75(7):E433–43. 10.1111/j.1750-3841.2010.01768.x 21535537

[23] Huang F, Fang Z, Mast J, Chen W. Comparison of membrane electroporation and protein denature in response to pulsed electric field with different durations. Bioelectromagnetics. 2013 May;34(4):253–63. 10.1002/bem.21773 23322376

[24] Keiller DR, Mackerness SA, Holmes MG (2003). The action of a range of supplementary ultraviolet (UV) wavelengths on photosynthesis in Brassica napus L. in the natural environment: effects on PS II, CO(2) assimilation and level of chloroplast proteins. Photosynth Res.

[25] Mildažienė V, Aleknavičiūtė V, Žūkienė R, Paužaitė G, Naučienė Z, Filatova I, Lyushkevich V, Haimi P, Tamošiūnė I, Baniulis D. Treatment of Common Sunflower (Helianthus annus L.) Seeds with Radio-frequency Electromagnetic Field and Cold Plasma Induces Changes in Seed Phytohormone Balance, Seedling Development and Leaf Protein Expression. Sci Rep. 2019 Apr 23;9(1):6437. 10.1038/s41598-019-42893-5 PMC6478675 31015543

[26] Carrión VJ, Perez-Jaramillo J, Cordovez V, Tracanna V, de Hollander M, Ruiz-Buck D, Mendes LW, van Ijcken WFJ, Gomez-Exposito R, Elsayed SS, Mohanraju P, Arifah A, van der Oost J, Paulson JN, Mendes R, van Wezel GP, Medema MH, Raaijmakers JM. Pathogen-induced activation of disease-suppressive functions in the endophytic root microbiome. Science. 2019 Nov 1;366(6465):606–612. 10.1126/science.aaw9285 31672892

[27] Soldan R, Mapelli F, Crotti E, Schnell S, Daffonchio D, Marasco R, Fusi M, Borin S, Cardinale M. Bacterial endophytes of mangrove propagules elicit early establishment of the natural host and promote growth of cereal crops under salt stress. Microbiol Res. 2019 Jun-Aug;223–225:33–43. 10.1016/j.micres.2019.03.008 31178049

[28] Wang M, Eyre AW, Thon MR, Oh Y, Dean RA. Dynamic changes in the Microbiome of Rice during shoot and Root Growth Derived from Seeds. Front Microbiol 2020 Sep 8;11:559728. 10.3389/fmicb.2020.559728 PMC7506108 33013792

[29] Durán P, Thiergart T, Garrido-Oter R, Agler M, Kemen E, Schulze-Lefert P, Hacquard S. Microbial interkingdom interactions in roots promote Arabidopsis Survival. Cell 2018 Nov 1;175(4):973–983e14. 10.1016/j.cell.2018.10.020 PMC6218654 30388454

[30] Ventorino Valeria,Sannino Filomena,Piccolo Alessandro et al. Methylobacterium populi VP2: plant growth-promoting bacterium isolated from a highly polluted environment for polycyclic aromatic hydrocarbon (PAH) biodegradation. ScientificWorldJournal et al. 2014, 2014: 931793. 10.1155/2014/931793 PMC4135167 25152928

[31] Wang Q, Ge C, Xu S, Wu Y, Sahito ZA, Ma L, Pan F, Zhou Q, Huang L, Feng Y, Yang X. The endophytic bacterium Sphingomonas SaMR12 alleviates Cd stress in oilseed rape through regulation of the GSH-AsA cycle and antioxidative enzymes. BMC Plant Biol. 2020 Feb 6;20(1):63. 10.1186/s12870-020-2273-1 PMC7006384 32028891

[32] Coutinho TA, Venter SN. Pantoea ananatis: an unconventional plant pathogen. Mol Plant Pathol. 2009 May;10(3):325–35. 10.1111/j.1364-3703.2009.00542.x PMC6640510 19400836

[33] Moon CD, Zhang XX, Matthijs S, Schäfer M, Budzikiewicz H, Rainey PB. Genomic, genetic and structural analysis of pyoverdine-mediated iron acquisition in the plant growth-promoting bacterium Pseudomonas fluorescens SBW25. BMC Microbiol 2008 Jan 14;8:7. 10.1186/1471-2180-8-7 PMC2235872 18194565

[34] Liu T, Burritt DJ, Oey I. Understanding the effect of Pulsed Electric Fields on multilayered solid plant foods: bunching onions (Allium fistulosum) as a model system. Food Res Int. 2019 Jun;120:560–7. 10.1016/j.foodres.2018.11.006 31000272

[35] Cooper RE, Wegner CE, Kügler S, Poulin RX, Ueberschaar N, Wurlitzer JD, Stettin D, Wichard T, Pohnert G, Küsel K. Iron is not everything: unexpected complex metabolic responses between iron-cycling microorganisms. ISME J. 2020 Nov;14(11):2675–90. 10.1038/s41396-020-0718-z PMC7784907 32690937

[36] Gu S, Wei Z, Shao Z, Friman VP, Cao K, Yang T, Kramer J, Wang X, Li M, Mei X, Xu Y, Shen Q, Kümmerli R, Jousset A. Competition for iron drives phytopathogen control by natural rhizosphere microbiomes. Nat Microbiol. 2020 Aug;5(8):1002–10. 10.1038/s41564-020-0719-8 PMC7116525 32393858

[37] Angelé-Martínez C, Goodman C, Brumaghim J. Metal-mediated DNA damage and cell death: mechanisms, detection methods, and cellular consequences. Metallomics. 2014 Aug;6(8):1358–81. 10.1039/c4mt00057a 24788233

[38] Vadivukkarasi P, Bhai RS. Phyllosphere-associated Methylobacterium: a potential biostimulant for ginger (Zingiber officinale Rosc.) Cultivation. Arch Microbiol. 2020 Mar;202(2):369–75. 10.1007/s00203-019-01753-6 31673721

[39] Meena KK, Bitla UM, Sorty AM, Singh DP, Gupta VK, Wakchaure GC, Kumar S. Mitigation of salinity stress in wheat seedlings due to the application of Phytohormone-Rich Culture Filtrate Extract of Methylotrophic Actinobacterium Nocardioides sp. NIMMe6. Front Microbiol. 2020 Sep 18;11:2091. 10.3389/fmicb.2020.02091 PMC7531191 33071995

[40] Xu J, Kloepper JW, Huang P, McInroy JA, Hu CH. Isolation and characterization of N2 -fixing bacteria from giant reed and switchgrass for plant growth promotion and nutrient uptake. J Basic Microbiol. 2018 May;58(5):459–71. 10.1002/jobm.201700535 29473969

[41] Maglangit F, Alrashdi S, Renault J, Trembleau L, Victoria C, Tong MH, Wang S, Kyeremeh K, Deng H. Characterization of the promiscuous N-acyl CoA transferase, LgoC, in legonoxamine biosynthesis. Org Biomol Chem 2020 Mar 25;18(12):2219–22. 10.1039/d0ob00320d 32159577

